# Memorization strategy and foreign language learning: a narrative literature review

**DOI:** 10.3389/fpsyg.2023.1261220

**Published:** 2023-09-12

**Authors:** Qunfeng Wang

**Affiliations:** English Department, School of Foreign Languages and Humanities, Xi’an University of Technology, Xi'an, China

**Keywords:** memorization strategy, vocabulary memorization, text memorization, EFL leaners, language learning strategy

## Abstract

Memorization strategy as a subset of language learning strategy (LLS) has long been investigated to explain foreign language learners’ learning behaviors and uncover the role that it plays in foreign language learning (FLL). In the past half century, the focus of memorization research in FLL has been shifted from Memorization, Vocabulary Memorization to Text Memorization, which are termed with consideration of the length of language material to memorize. Since memorization strategy use is greatly influenced by varieties of psychological and socio-culture variables, the memorization strategy system becomes more complicated in the process of FLL. The present narrative review attempts to provide an overview of memorization strategy research in the field of FLL by discussing the concepts, categorizations, uses, instructions and influential factors of the three types of memorization strategies. By reviewing the existing studies, this paper proposes that in future research, diversified methods be deployed with the expansion of research perspectives and the enrichment of research topics to reveal the relationship between memorization strategy and FLL more extensively.

## 1. Introduction

Learning strategy is perceived as one of the influential factors relating to learner’s learning outcomes. In the field of FLL, particularly learning English as foreign language (EFL), efforts have been made in the previous research to explore the relationship between LLS and FLL with a systematic identification of varieties of strategies that EFL leaners deploy in the process of language learning (O’Malley and Chamot, 1990; Macaro, 2001; Oxford, 2017; Chamot and Harris, 2019). Among different groups of LLSs, memorization, memorization strategy, or memory strategy, has been valued since it is considered as the initial step in learning a language in a non-native language speaking context.

With the introduction of cognitive theory to FLL, memorization is treated as a part of the cognitive learning process. A number of studies have been focused on the classification of memorization with discovery of specific memorization strategies employed by foreign language learners and their contributions to FLL. With the expansion of the scope of LLS into investigations of strategies in relation to specific language skills, due attention, in further studies, has been paid to associate memorization with language skill areas, such as speaking and writing (He and Shi, 2008; Chen et al., 2016). In these studies, memorization refers to the strategies to commit the language material, short or long, to memory with little consideration of its length. However, some scholars find that many of these strategies are closely associated with vocabulary learning (Takač, 2008; Gu, 2010; Sinhaneti and Kyaw, 2012). Afterwards, vocabulary memorization strategy or memorization as vocabulary learning strategy starts to be used as a more concrete and specialized terminology to describe the strategies to memorize words, the smaller language units. A number of studies have been conducted to explore the relevant issues like vocabulary memorization strategy use and instruction. Rather than being focused on the impact of memorization and vocabulary memorization on FLL, the more recent research has shed light on text memorization, suggesting a conspicuous division between the concept of vocabulary memorization strategy and text memorization strategy in the hope of uncovering how EFL learners treat the long and consecutive texts in the process of memorization to facilitate their language learning (Harris, 2015; Yu, 2017; Wang, 2023). Therefore, memorization strategy can be historically sorted into three types, namely memorization, vocabulary memorization and text memorization, when the length of language material for memorization is considered.

Narrative literature review can present a comprehensive background for a particular topic by synthesizing a broad spectrum of the literature into a coherent interpretation that highlights the major issues, trends, complexities, and controversies that are at the center of it (Efron and Ruth, 2019). Together with consideration of the longstanding history of memorization research, narrative literature review is adopted in this paper. Only published scientific research articles that are written in English and relevant to memorization, vocabulary memorization and text memorization in the field of FLL are considered for the review. Conference and review papers are not included. The present narrative review aims to go through the studies concerning the three types of memorization strategies and their relationships with FLL, summarize the development and major findings in the past half century and offer directions for future research on memorization as a foreign LLS.

## 2. Memorization in FLL

### 2.1. Concept of memorization

Memorization strategy, historically named mnemonics, is also termed memory strategy or simply memorization in the existing literature. Oxford (1990) defines it as strategies that help learners store and retrieve new information. Since it is a fundamental part of the learning process with involvement of storage and retrieval of what has been learned, the value of memorization, as one consequential subcategory of LLS, can be prominently seen in the definitions of learning strategy by many researchers. The role of memorization is portrayed in an early definition by Dansereau (1985), who regards learning strategy as the operations that leaners deploy for acquisition, *storage*, retrieval, or use of information and intentional behavior. Richards and Platt (1992) refer to memorization as thoughts used by learners during learning to better help understand, learn or *remember* new information. “Storage” and “remember” can be taken as the synonyms of memorization in a broad sense, or at least the similar concept in meaning that highlights the information input in the cognitive process in learning. With the introduction and extensive application of cognitive theory into FLL, the significance of memorization in language learning is identified by defining foreign LLS as strategies that help learners take in various aspects of the language, *store them in long-term memory*, and use them when needed (Okada et al., 1996) and teachable actions that learners choose from among alternatives and employ for L2 learning purposes, e.g., constructing, internalizing, *storing*, retrieving, and using information (Oxford, 2011). Likewise, Cohen (2014) correlates memorization with LLS for formally *committing to memory* whatever material is not acquired naturally through exposure. Though some views on memory strategy are not positive, memory-related words, such as “remember” and “store” with other linguistic forms like “storage” and “storing,” are employed to describe and define learning strategy or LLS. To foreign language learners, for instance, EFL learners, as they are not able to gain many opportunities to be exposed to an English-speaking environment, memorization strategy can be a useful aid in the learning process to a large extent. So the significance of memorization as a part of the cognitive process in FLL is evident.

### 2.2. Classification of memorization

Though language scholars hold different views on the categorization of LLS, many of them have highlighted the importance of memorization as a foreign LLS when groups of sub-memorization strategies facilitating language learning are investigated. Rubin (1981) notes that storage and retrieval of information are involved in language learning, so memorization, by which language materials are stored, stays a vital part. As a basic subset of strategies that directly affect learning, such strategies as to take note, pronounce out loud, find association and use mechanical devices appear in his memorization strategy list. The most outstanding contribution to the classification of memorization is made by Oxford (1990), stressing that memory strategy is a mighty mental tool for learners to not only store but retrieve language information. In detail, it is composed of four categories: creating mental linkages, applying images and sounds, reviewing well, and employing actions, which are further divided into 10 subsets. Though Cohen (2014) does not make a clear-cut classification of memory strategies, all the subsets of language learning strategies presented by him, including strategies for identifying, distinguishing, grouping and contacting repeatedly with learning materials, seem to contribute to memorizing the language materials that are not acquired by natural exposure. In fact, the memorization classification is accompanied with the taxonomy of LLS as a result of the enquires into how language learners process, store, and retrieve language learning materials. No matter how memorization strategy is classified, as a powerful mental instrument for storage and retrieval of language information, the role of memorization in the language learning process can never be overlooked.

### 2.3. Memorization and understanding

The endeavor to investigate information processing in cognitive psychology leads to the recognition of memorization as a fundamental learning strategy in the learning process. A number of studies, not excluding the ones in the field of FLL, reveal that the adoption of memorization strategy is beneficial to enhance learning (Kember, 1996; Hulstijn, 2000; Alieh and Atefeh, 2015). But for years, memorization has been regarded as being mechanic and a barrier to prevent creativity in learning, so such strategy is not encouraged and receives criticism particularly in western settings. However, the fact that those with Asian backgrounds, such as Chinese, Japanese and Korean, become successful learners by heavy reliance on memorization prompts scholars to uncover the paradox (Mok et al., 2001; Chan and Rao, 2009). Researchers suggest the variation between rote memorization and meaningful memorization which consists of two forms: understanding followed by memorization; memorization and understanding work simultaneously (Marton et al., 1996). This finding is echoed by Mugler and Landbeck (2000) who classify memorization into two groups: rote learning that implies a lack of understanding, and memorization that implies understanding. On the basis of the sequential order of understanding and memorization, three models are unfolded: memorization, then understanding; understanding, then memorization; combination of both. These are termed, respectively, by Marton et al. (2005) rote memorization, in which memorization precedes understanding, meaningful memorization 1, in which memorization succeeds understanding, and meaningful memorization 2, in which memorization and understanding are combined to facilitate leaning at the same time. A similar finding that memorization does not function alone, but works with understanding in the Asian learners’ learning process is obtained in a case study involving 21 Chinese students studying in UK (Mathias et al., 2013). In other studies, the distinction between good memorization and bad memorization is proposed with opposition to bad memorization use because it is good memorization with understanding that helps internalize what has been learned. (Oanh and Hien, 2006; Khamees, 2016). To conclude, memorization does not necessarily mean rote learning that is traditionally defined as mechanical learning only by repetition. When understanding is engaged in the process of memorization, memorization strategy can be ultimately positive and advantageous to language learning.

### 2.4. Memorization use and instruction

Since language learners are not often rote or passive learners when meaningful or good memorization strategy is applied, many researchers find that memorization has positive effects on language learning (Li, 2012; Hashim, 2015). In many studies (Hong-Nam and Leavell, 2007; Sung, 2011; Ranjan et al., 2021), the role that memorization plays in FFL is investigated by using Oxford’s Strategy Inventory for Language Learning (SILL) or other questionnaires concerning memorization use. For example, by application of SILL, together with a semi-structured interview, an open-ended questionnaire and the respondents’ online interactions in a language task, the study by Shakarami et al. (2011) finds that the Malaysian Net-Generation frequently uses memorization strategy with a proposal to re-conceptualize the notion of memorization strategy in SILL to cope with learner’s needs. In another study (Khamees, 2016), a questionnaire is administrated to 66 FLL learners to uncover which language aspect is most frequently memorized and the reasons for memorization strategy use. The findings suggest that memorization facilitates language learning and improve learners’ confidence, but its use is among some limited areas of language, such as vocabulary, term definitions and literary extracts. In addition, it is pointed out that rote memorization needs to be avoided.

With the discovery of the favorable impact that memorization imposes on FLL, how the specific memorization strategies can be applied to the areas of foreign language skills also arouses researchers’ interest. Oxford (1990) demonstrates how each of the ten subsets of memory strategies helps develop language learners’ four language skills (listening, reading, speaking and writing) with a focus on elaboration of storage function of each memory strategy. In a questionnaire-based study, Ozkan and Kesen (2008) make a comparison between memorization use in speaking and writing, reporting that both freshmen and seniors tend to employ memorization to improve their writing and speaking skills, but do it more often in writing than speaking. A research with an experimental group and a control group is conducted by Chen et al. (2016) to explore the effects of memorization use on improvement of EFL learners’ oral English proficiency. The findings indicate a significant difference in learners’ speaking accuracy and fluency. The experimental group who acquires memorization by class instructions performs better than the controlled group with no instructions.

One study, conducted by He and Shi (2008) with an attempt to make inquiries about the performances of 16 interviewed Chinese students studying at a Canadian university in two English writing tests, is worthy to be underscored. In the study, many Chinese participants pass Test of Written English, an entrance writing test for ESL/EFL learners, by dependence on memorization of the writing samples, but fail in English Language Proficiency Index (LPI) for both ESL/EFL learners and some native speakers. Many of them attribute their success to the pre-test training focused on memorization in China and complain LPI training course centered on developing writing skills in Canada. Thus, sociocultural factor beyond LLS itself, is involved in language learning, so memorization use becomes a culture-related issue. As an individual difference, memorization can be more likely individualized and affected by cultural and social factors. So, it is necessary and of significance to consider other individual differences when memorization is investigated.

## 3. Vocabulary memorization in FLL

### 3.1. Concept of vocabulary memorization

The original intention of studies on memorization by psycholinguists and applied linguists is to inquire into the strategies that language learners deploy to memorize words, formulaic sequences (FSs), frequently-used simple sentences, basic sentence patterns, dialogues or monologues to ameliorate language learning (Rubin, 1981). The materials for memorization include both short and long language units, so the term “memorization” in the early studies is used in a boarder sense and seems to be more general. Many subgroups of memorization strategy concluded in the early memorization list, such as note-taking, associations, keyword, structured-reviewing etc. (Cohen and Aphek, 1980; Pressley et al., 1982; O’Malley and Chamot, 1985; Oxford, 1990) are recognized as strategies that work mostly to memorize vocabulary, the smaller lexical items. With scholars’ increasing interest on vocabulary learning strategy, this issue is later noticed and leads to the use of a more accurate terminology to refer to the strategies to memorize vocabulary. However, an agreement on the terminology has not been reached since varieties of terms are being used, such as vocabulary memorization strategy, memorization as a vocabulary learning strategy, memory strategy for vocabulary learning etc. Among them, vocabulary memorization is used in the present review and suggested to be used in the future research because of its simplicity and clarity.

### 3.2. Vocabulary memorization as a subset of vocabulary learning strategy

Similar to studies on general memorization discovered with the taxonomies of LLS, the research on vocabulary memorization develops with the categorization of vocabulary learning strategy. Vocabulary learning strategy is firstly put forward by Stoffer (1995), which consists of 9 groups based on the data collected from Vocabulary Strategy Inventory. Memorization by repetition and memorization by coding are concluded as two important strategies in the typology of vocabulary learning strategy by Gu and Johnson (1996), which corresponds to 91 statements of Vocabulary Learning Questionnaire devised. In Vocabulary Learning Strategies Questionnaire (VLSQ) designed by Schmitt (1997), 27 strategies form memory strategy and other 31 strategies fall into cognitive, metacognitive and social strategy. This can be considered as the extraction and continuity of Oxford’s classification of LLS and the most comprehensive typology of vocabulary learning strategy. In a recent study on the taxonomy of vocabulary learning strategy by Gu (2018), 62 specific vocabulary learning strategies fall into two dimensions: metacognitive and cognitive. Among the cognitive, many strategies, for example, using dictionary, rehearsal and encoding, contribute to memorizing words. As what he points out, a wide array of vocabulary learning strategy are used to commit words to memory at different stages of language learning (Gu, 2019). However, the classification of vocabulary learning strategy has also stirred up controversy because one strategy may fall into more than one group. In terms of sub-vocabulary memorization strategy, the taxonomies are sometimes inconsistent depending on the functions or purposes of a strategy in distinctive contexts. For example, verbal repetition is categorized into memorization strategy in Gu and Johnson’s typology, but cognitive strategy in Schmitt’s. Despite of such problem, the taxonomy of vocabulary learning strategy is helpful to not only extend the scope of LLS research, but also shift the focus of memorization studies from the more general memorization strategy to the specialized memorization strategy, memorization as a vocabulary learning strategy.

### 3.3. Vocabulary memorization use and instruction

The first focus of vocabulary memorization studies is about the frequency of vocabulary memorization use and how it works to better foreign language learners’ vocabulary learning. The research is often conducted by applying Schmitt’s questionnaire VLSQ as well as other questionnaires adapted from the previous ones, like Oxford’s SILL. Memory strategies are found to be frequently used by language learners to contribute most to vocabulary achievements in some studies (Kafipour et al., 2011; Kocaman et al., 2018), whereas other studies report that vocabulary memorization strategy is used at a medium level, or even a low level (Amirian and Heshmatifar, 2013; Kesmez, 2021). Moreover, attention has been given to the frequency of the sub-vocabulary memorization strategy use. Al-Qaysi and Shabdin (2016) examine the Arab students’ vocabulary memorization strategy use of four groups, namely creating mental linkages, applying images and sounds, reviewing well, employing actions, together with 25 specific vocabulary memorization strategies. It is reported that the most preferred strategy is reviewing well while employing actions is the least frequently used strategy. Besides, repetition is most favored among the subgroups of vocabulary memorization strategy because it takes effects in enhancing vocabulary retention. Based on VISQ and a semi-structured interview, Aravind and Rajasekaran (2019) reveal that Indian learners regard vocabulary memorization as an important strategy to restore and retain vocabulary. Among 25 vocabulary memorization strategies examined, the top three are to use the new word in sentences, study word with a pictorial representation of its meaning and connect word to a personal experience. The inconsistent findings about frequency use of memorization strategy and variations on sub-vocabulary memorization strategy choices by language learners might be caused by the sample size as well as the differences in participants’ age, sex and cultural backgrounds etc., which are assumed to be the potential factors that influence learners’ vocabulary memorization strategy use.

In addition to the research mostly based on questionnaires and interviews, some quantitative studies have focused on whether vocabulary memorization strategy can be learned through instructions to benefit vocabulary acquisition. The findings of the prior research seem to be consistently positive though the issue of whether LLS can be taught effectively, learned successfully and then deployed purposely by language learners are not uncommonly debatable. These studies, which intend to evaluate the effectiveness of vocabulary memorization strategy teaching, are normally conducted by instructions of a group of vocabulary memorization strategies, for instance, grouping, contextual effect, and imagery, with the engagement of a control group and an experimental group. Nemati (2009, 2010) finds that vocabulary memorization strategy instructions are productive because learners who are taught vocabulary memorization strategies outperform the learners who receive no instructions both in an immediate test and a delayed test. The results suggest that the instruction is helpful for both short-term and long-term vocabulary retention, which is in line with the findings of Marefat and Shirazi (2003) indicating the effectiveness of vocabulary memorization strategy instructions. But, Ghorbani and Riabi (2011), with the adoption of a similar instruction procedure, report that vocabulary memorization strategy instruction only facilitates the process of long-term EFL vocabulary retention, and its impact on short-term retention is not significant. There are also other studies that focus on the relationship between instruction of a certain vocabulary memorization strategy, such as key word method (Tavakoli and Gerami, 2013), sematic mapping (Badr and Abu-Ayyash, 2019), association (Fatemeh and Ghaffar, 2012), and vocabulary learning achievements. The results consistently show that instructions are effective to promote vocabulary retention. To conclude, the existing literature pinpoints that instructions of vocabulary memorization strategy take effect on improvement of foreign language learners’ vocabulary learning in spite of the discrepancy on short-term and long-term retention of vocabulary, which might be attributed to the sub-vocabulary memorization strategy selected or materials used for the instructions. However, when EFL learners acquire one vocabulary memorization strategy or a set of vocabulary memorization strategies by being trained, it does not necessarily mean that they would intend to employ them in learning due to personal preferences, social contexts or other factors. Therefore, more studies on the issue of instruction, learning and use of vocabulary memorization strategy with consideration of multiple variables are needed in the future research.

## 4. Text memorization in FLL

### 4.1. Concept of text memorization

As noted above, the majority of the existing research on memorization and vocabulary memorization has been much centered on memorization of the smaller language units. But, when the material for memorization is extended from words to longer consecutive texts, the memorization process can possibly alter and become more complicated. Since text memorization is considered to be a traditional and effective learning practice in Asian countries (Yu, 2013a), learners are encouraged to memorize the texts, for example, sentences, passages and the entire articles when learning a foreign language. The exclusion of the more complex language material in memorization studies and the Asian foreign language learners’ success achieved by reliance on reciting textual materials have prompted, in the past recent years, some scholars to investigate the differentiations of memorization procedures resulted from the changes of the length of language materials to memorize. Therefore, in order to distinguish the early memorization and vocabulary memorization, a more specialized terminology, text memorization, is used in the literature to particularly define foreign language learners’ strategic behavior to memorize longer linguistic components generalized as texts.

### 4.2. Text memorization use

The early observation correlating memorization to text is made by Pennycook (1996), who points out the role of acceptable borrowing through memorization of texts in FLL by looking at learning in a Chinese context. The successive studies on text memorization use have focused on three respects. With regard to the gains brought by text memorization (Ding, 2007), by the interview with three winners in the English speaking and debate contest at the national level in China, it is reported that the practice of text memorization and imitation helps language learners attend to and learn collocations and sequences, which can be borrowed for language production. In addition, it enables leaners to make improvement on pronunciation and develop the habit of attending to the details of language. Finally, it is concluded that noticing and rehearsal can be enhanced to be beneficial to FLL. Based on a semi-structured interview administrated to 62 students and teachers from junior, senior high school and college, the qualitative findings from Yu (2013b) reveal that text memorization could facilitate language learning psychologically in three aspects: cultivation of “language sense,” establishment of conscious learning, and increase of psychological satisfaction built on learners’ sense of achievement and confidence. And more specifically, it could help improve the learning of phrases, sentence structures and grammar, develop writing and speaking skills, and enhance vocabulary learning in the contexts. Besides qualitative analysis, a few quantitative studies have been conducted to reveal the effectiveness of text memorization. Dai and Ding (2010) find that compared to learners without practicing text memorization, learners from the experimental group who practice text memorization perform better in both English proficiency test and writing test. Moreover, text memorization enables the high achievers to use FSs, the multiword expressions that occur frequently as coherent semantic units, more accurately and the low achievers to learn to use more FSs in a broader range of variety.

### 4.3. Text memorization procedure

In terms of how language learners proceed with text memorization, some attempts are made in the prior studies. One potential concern for text memorization is the selection of the texts that are suitable to memorize. Harris (2015) proposes such criteria consisting of five items: appropriateness of difficulty level, stimulating and memorable content, concrete content and clear writing, a natural and logical flow in the text, the presence of rhythm and/or rhyme. Furthermore, in order to ease the task of text memorization, a systematic method including the top-down strategy and the bottom-up strategy to analyze the articles for memorization is depicted. The top-down strategy requires the examination of the overall content, according to which four steps need to be followed: read through the texts, focus on the main idea, structure establishment and analysis of each sentence for general idea. Whereas the bottom-up involves the analysis at lexical level, for instance, identification of formulaic chunks and core words, and to note how the words are combined to create meaning.

As to what specific strategies are deployed in the process of text memorization, some frequently used sub-strategies for text memorization, such as imitation (Ding, 2007), recitation (Yu, 2017), and reading aloud (Yu and Liu, 2018), are pointed out in the previous literature. In a more recent study, Wang (2023) develops a new system of text memorization strategies based on the analysis of Chinese EFL learners’ text memorization process and strategies deployed. So far, studies to present a system of text memorization strategy are limited. However, it is necessary to treat a variety of text memorization strategies in a systematic manner and categorize them into different groups. Though the classification might also be questioned as the taxonomy of memorization and vocabulary memorization has been criticized, it would come to the aid of foreign language learners to be aware or conscious of these strategies to benefit their learning.

To conclude, a common and noticeable merit of text memorization discovered in the literature lies in the opportunities it offers for learners to memorize FSs in the meaningful contexts. Moreover, text memorization is often associated with text analysis for understanding which in turn reinforces the memorization effects. To date, the majority of studies on text memorization as an FLL strategy are conducted by Asian scholars probably in that it is culturally oriented. It is assumed that like vocabulary memorization, text memorization can also be taught and learned, but studies on text memorization instruction can be rarely found and need to be explored in the future.

## 5. Influential factors of memorization use

Griffiths and Soruç (2020) underpin that all the individual variables be examined in a holistic way since one variable could rely on another or more to construct a complex, dynamic and situated system to be influential to language learning. Therefore, memorization strategy can interact with many other factors to influence learners’ language development.

The findings about memorization use and FLL learners’ language proficiency in the existing documents are comparatively consistent. The comparison is normally made between beginner (lower level) and advanced (higher level) learners and sometimes the intermediate learners are also concerned. It is revealed in many studies that the more proficient FLL learners use less memorization strategies than the less proficient learners and the role that memorization strategy plays in language learning seems not as significant as other LLSs to the more proficient learners (Shmais, 2003; Gharbavi and Mousavi, 2012). The advanced foreign language learners tend to favor non-memory strategy, for example, metacognitive strategy rather than memorization strategy to plan and regulate their language learning process (Lee and Heinz, 2016; Lin, 2017). Therefore, an inverse relationship between language proficiency and memorization strategy use has been found. Language proficiency has become an important factor that influences learners’ memorization strategy use.

Among all the potential variables (e.g., age, sex, belief etc.), culture has been extensively explicated as being associated with memorization stagey use. Traditionally, it seems recognized that foreign language learners from the culture greatly influenced by Confucianism, mostly Chinese, Korean and Japanese, prefer to deploy memorization as their major LLS, which is supported by a succession of studies concerning memorization use (Biggs, 1996; Cortazzi and Jin, 1996; Tan, 2011). However, the findings from many of the more recent studies reveal that not only learners with Confucian background give priority to memorization use in the FLL process, but also learners with a little or no Confucian influence frequently make the most of memorization strategy and find it efficient to strengthen their FLL learning. For example, Pakistani EFL learners’ memorization strategy is reported to be more significant than social and affective strategy in a survey-based study (Khan et al., 2018). By interviewing 18 English learners, the findings reveal that memorization strategy is frequently used by Indonesian EFL learners and it is helpful to improve their English proficiency (Alfian, 2021), which mirrors Rianto’s (2020) study suggesting that Indonesian learners rely on memorization strategy highly. Moreover, some studies, inquiring into the cognitive language learning process of CFL (Chinese as a foreign language) learners, find that European and American CFL learners, similarly, take advantage of memorization strategy in their learning process (Jiang and Cohen, 2012). For example, in the study involving 190 English students, Grenfell and Harris (2015) explore the memorization strategies used by English CFL learners and conclude that these strategies are beneficial to their Chinese learning. Therefore, memorization strategy is not only the preference of learners influenced by Confucian philosophy, but of learners with other cultural backgrounds. However, the low use of memorization strategy by students with a little or no Confucian background is also found in a few other studies (Magno, 2010; Alhaisoni, 2012). When culture is taken into account, the results about memorization use in FLL remains contradictory and thus confusing, which makes the issue of memorization use more complicated. Such controversy might lie in that culture is not always the only factor influencing learners’ strategy use, but other factors like personality and age would work together. Therefore, a holistic view needs to be taken to further interpret memorization strategy use in language learning.

Thus far, only a few studies on how other variables such as age, sex and belief interact with memorization strategy to influence FLL can be found. In terms of age and sex, the findings of study by Jiang and Smith (2009) suggest that both two variables do not significantly influence the frequency of memorization use in FLL. In their research, based on an investigation into 13 Chinese EFL learners aged from 8 to 40, it is found that all the participants report much use of memorization though some specific strategies to learn vocabulary and grammar and the frequency of use of mother tongue differ. With regard to the relationship among belief, memorization use and learning achievement, Rashidi and Omid (2011) conclude that Iranian EFL learners’ beliefs and memorization strategy use is positively correlated, but there is no significant association between learners’ beliefs about rote memorization and their learning achievements.

To summarize, varieties of factors that influence memorization strategy use have been investigated, such as FLL learners’ language proficiency, culture, age, sex and belief. But how memorization strategy functions with other individual differences to impact language learning remains unknown. Therefore, the perception of multiple interactions of memorization with other variables should be taken in future research to provide more evidence on memorization use.

## 6. Suggestions for future research

The past 50 years’ research on memorization strategy in FLL can be concluded to experience three phases with different focuses: the initial studies on memorization, the following studies on vocabulary memorization and then the extended studies on text memorization. Based on a review of the previous achievements made and the problematic issues diagnosed, some suggestions for future research on memorization and FLL are proposed.

The research perspectives need to be expanded. First, memorization strategy use is contextually situated, so it is impacted by foreign language learners’ sociocultural environment. Memorization can also interact with many other individual differences, such as motivation, age, sex etc., to influence language development. Moreover, in practice, memorization strategies seldom function in isolation, but in sequences or clusters. Language learners tend to employ varieties of sub-memorization strategies and other LLSs simultaneously or in an overlapping approach to increase learning effectiveness. Therefore, memorization strategy in FLL is suggested to be examined with the introduction of the theory of social-ecological context and holism. Second, though FLL and SLL (second language learning) share some similarities in the learning procedures, FLL and SLL learners’ memorization use could differ. For example, when a group of Chinese EFL learners move from China to Singapore in an ESL setting, great changes in task demands and input/output opportunities are brought out because of the changes of the learning contexts (Gu, 2010). Chinese EFL learners have to employ more effective memorization strategies in a non-English speaking context because opportunities for them to be exposed to English are limited. To EFL learners, learning English in a natural language environment is barely possible. So it is strongly suggested to draw a clear division line between memorization strategy use in FLL and SLL while memorization strategy is investigated. Third, when the materials to memorize extend from short language units to longer language elements, the memorization process and the sub-memorization strategies involved could alter accordingly. As a result, the length of the materials for memorization should be considered carefully when studies of memorization are conducted.

More research topics can be suggested. Many previous studies have mostly focused on correlating choice of memorization strategy (including the early memorization, vocabulary memorization and text memorization) to culture or learners’ language proficiency. In future studies, the relationship between FLL and other potential variables, for instance, personality, interest, and motivation, needs to be explored so as to provide a more holistic insight into language learners’ memorization use. Furthermore, in terms of vocabulary memorization instruction and learning, future research could be concerned about to what extent learners are willing to use memorization strategies after they are successfully acquired. After all it is a sort of personal preference not independent on one’s learning style and other differences. In addition, with regard to text memorization, a variety of issues could be further researched. First, since the process of text memorization becomes more complicated with longer materials and more sub-strategies involved, researchers need to reveal the procedure of memorizing these materials, that is, how learners treat longer language elements to increase memorization effectiveness to facilitate their language learning. Second, series of sub-strategies of text memorization need to be presented with a systematic taxonomy in the future studies to enable language learners to learn and put them into practice to enhance their language learning. Third, theoretically, with the accumulation and storage of more consecutive language materials, language learners are able to retrieve language information when needed in the specialized language areas, but evidence that supports this assumption are not adequate. Therefore, more attention can be paid to uncover the role that text memorization plays in improving learners’ language skill levels, especially that of speaking and writing. Fourth, the issue about whether text memorization strategies can be instructed, learned and then employed by language learners is also worthy of being researched.

Research methodologies are suggested to be more diversified. Many researchers have designed either qualitative or quantitative research to discover the relationship between memorization and FLL. The quantitative methods are concerned with the whole group of language learners’ average response to memorization-related issues while the qualitative methods focus on subjective views and experiences of individuals. Since with the application of both qualitative and quantitative approaches, the quantitative and qualitative research strengths can be combined and the best of both paradigms can be brought out (Zoltán, 2007), it is suggested that more mixed methods research be developed so that the qualitative and quantitative data can be triangulated each other to improve the research validity. Moreover, in the previous studies, among a number of ways for quantitative data collection, the most widely-used instrument is the questionnaire and the intervention method is ignored. However, the experimental or quasi-experimental approach is significantly effective to tackle the problem about the relationship between memorization and FLL. Particularly, when the memorization outcome and its impact on FLL are examined and the effectiveness of memorization strategy instruction is explored, a comparison between the experimental group and the control group can provide more credible evidence. So, in the cases where the effects of initial group difference can be well controlled, more experimental or quasi-experimental research is suggested to be conducted.

## 7. Conclusion and limitations

This paper provides a narrative review on the historical development of research on memorization as an FLL strategy. Taken as a whole, the research on memorization and FLL in the past half century lies in three aspects according to the length of the linguistic materials to memorize: Memorization, Vocabulary Memorization and Text Memorization. In the review, the research status of the three types of memorization in FLL, with focus on their concepts, categorizations, uses, instructions and influential factors, are analyzed. The analysis on these themes and gaps in relation to memorization and FLL can be beneficial to the research in the future to better explain foreign language learners’ use of different types of memorization strategy and learning behaviors, thus facilitating their language learning. Since memorization as an FLL strategy and a cluster of psychological and socio-culture variables involved constitute a complex system, with the employment of diversified methods, the more extensive and in-depth research with focus on the enriched topics from different perspectives is needed in the future.

Although this narrative review provides new insights into research on memorization and foreign language learning, only the articles that center on English as a foreign language and a few articles that focus on Chinese as a foreign language are included. Moreover, the relevant articles reviewed are all published in English and non-English ones are not concerned. In addition, since text memorization is a comparatively new topic compared to memorization and vocabulary memorization in the field of foreign language learning, articles available to be reviewed are limited. Therefore, the results need to be cautiously interpreted when the relationship between memorization and other language as a foreign language and the association between text memorization and foreign language learning are researched. Future studies are suggested to include the literature that explores learners’ use of memorization in learning other language as a foreign language to offer more evidence to explain foreign language learners’ memorization use in the process of learning.

## Author contributions

QW: Writing – original draft, Writing – review & editing.

## Funding

The author(s) declare financial support was received for the research, authorship, and/or publication of this article. This work was supported by Social Science Foundation of Shaanxi Province, China (2019M006); Education and Teaching Reform Project of Xi’an University of Technology (xjy2143): 2021 Graduate Education and Teaching Reform of Xi’an University of Technology.

## Conflict of interest

The author declares that the research was conducted in the absence of any commercial or financial relationships that could be construed as a potential conflict of interest.

## Publisher’s note

All claims expressed in this article are solely those of the authors and do not necessarily represent those of their affiliated organizations, or those of the publisher, the editors and the reviewers. Any product that may be evaluated in this article, or claim that may be made by its manufacturer, is not guaranteed or endorsed by the publisher.
